# Posterior mediastinal leiomyosarcoma: Case report and literature review

**DOI:** 10.1097/MD.0000000000037704

**Published:** 2024-04-05

**Authors:** Hongzhen Zhao, Zhonghui Hu, Lingxin Kong, Qingtao Zhao, Wenbo Wu, Guochen Duan

**Affiliations:** a Hebei Medical University, Shijiazhuang, People’s Republic of China; b Department of Thoracic Surgery, Hebei General Hospital, Shijiazhuang, People’s Republic of China; c Department of Thoracic Surgery, Children’s Hospital of Hebei Province, Shijiazhuang, People’s Republic of China.

**Keywords:** leiomyosarcoma, mediastinal tumor, posterior mediastinum

## Abstract

**Background:**

Posterior mediastinal leiomyosarcoma is an extremely rare malignant mesenchymal tumor with no special clinical symptoms, which is easily confused with some common tumors in the posterior mediastinum, affecting the accuracy of the first diagnosis by clinicians and delaying the treatment of patients.

**Case summary:**

We report a 59-year-old woman with a space-occupying lesion in the posterior mediastinum. The patient was mistakenly diagnosed with lumbar muscle or vertebral body lesions due to chest and back pain and underwent conservative treatment, but her symptoms did not improve significantly and she gradually developed pain in both lower limbs. Chest computed tomography (CT) scan indicated the left lower lung paraspinal space and underwent standard single-aperture video-assisted thoracoscopic surgery (VATS), which was pathologically confirmed as posterior mediastinal leiomyosarcoma.

**Conclusion:**

Complete surgical resection of posterior mediastinal leiomyosarcoma can achieve good clinical results.

## 1. Introduction

Posterior mediastinal leiomyosarcoma is an extremely rare malignant mesenchymal tumor in clinic, with only a few cases reported. There are no obvious characteristics of clinical symptoms, which leads to difficulty in the diagnosis. Incorrect diagnosis would lead to delays and errors in treatment, and the prognosis is usually poor. We report a case of leiomyosarcoma of the left posterior mediastinum following single-aperture video-assisted thoracoscopic surgery (VATS).

## 2. Case report

**Figure 1.**
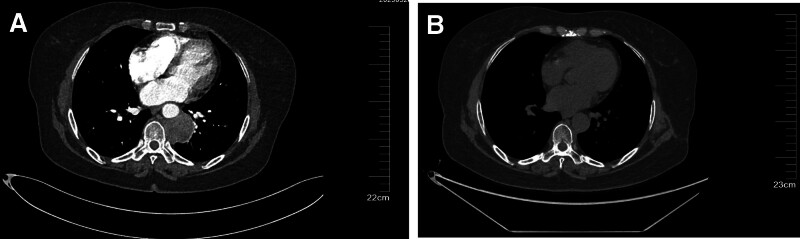
Imaging manifestations of patient. (A) CT manifestations paraspinal space in the lower left lung, which was closely related to the thoracic aorta. (B) CT manifestations of review. CT = computed tomography.

A 59-year-old female patient was admitted to Hebei General Hospital complaining of chest and back pain for 6 months. Before admission, the patient has misdiagnosed the pain as lumbar spine disease or muscle pain. After acupuncture and moxibustion and drug treatment, the symptoms did not improve significantly. And pain in both lower limbs eventually appeared. Chest computed tomography (CT) scan was performed in a local hospital, indicating paravertebral mass of the left inferior lung lobe. The patient had no chronic diseases such as hypertension, diabetes and coronary heart disease. After admission, the physical examination found that the patient had mild tenderness in the left chest and back, and the complete electrocardiogram examination showed no obvious abnormalities. A contrast-enhanced CT scan of the chest in our hospital showed paraspinal lesion of 46 × 41 × 41 mm in the posterior mediastinal, indistinguishable from the left inferior lung lobe (Fig. 1A). The lesion showed obvious uneven enhancement in the CT image, which boundary with the thoracic aorta was unclear, and ground glass shadow was visible around the edge of the lesion. The lesion was radiologically diagnosed as a neurogenic tumor.

**Figure 2.**
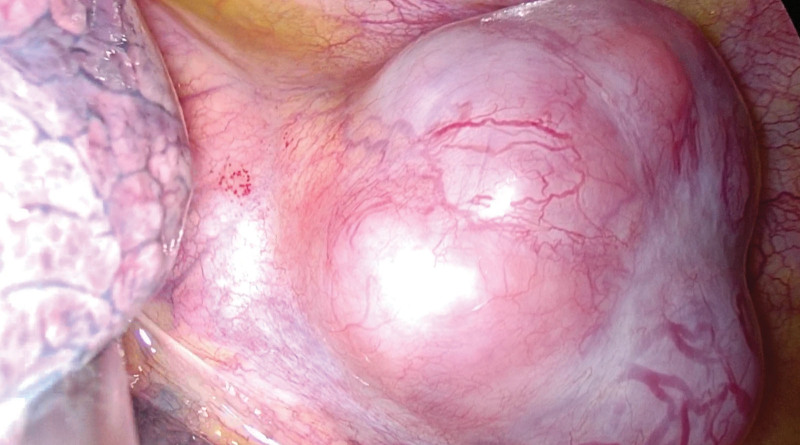
Diseased tissue was observed during the operation.

**Figure 3.**
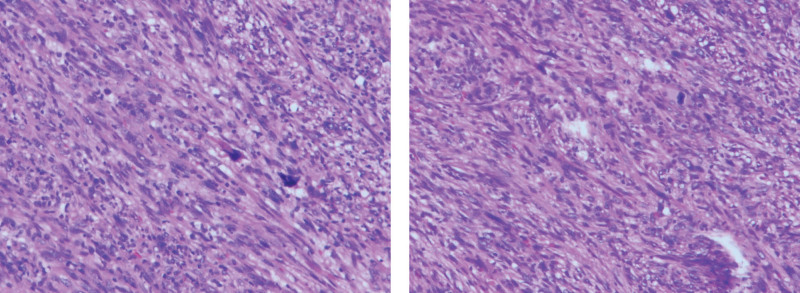
Histopathologic appearances of the tumor, spindle cell tumor 1 (H&E, ×40). Histopathologic appearances of the tumor, spindle cell tumor 2 (H&E, ×40).

After excluding surgical contraindications, the patient underwent VATS. The left fifth intercostal axillary line approach was taken. The tumor was found to be located in the posterior inferior mediastinum under thorascopic exploration. The lesion compressed the thoracic aorta and was close to the posterior chest wall and spine (Fig. 2). The tumor was completely resected during surgery, and part of the tissue was sent to rapid freezing pathology exams, which indicated spindle cell tumor (Fig. 3). Immunohistochemical staining of the complete lesion suggested leiomyosarcoma: CKpan (−), Vimentin (+), according to (−), SOX10 (−), SMA (+), NF (−), CR (−), Ki - 67 (active area about 40% +), Desmin (+), the EMA (−), P53 (−). The patient recovered well and was discharged on the sixth day after surgery. Postoperative radiotherapy was taken. The patient was reexamined by chest CT (Fig. 1B) at 1-month and 4-month after surgery and no recurrence was found.

## 3. Discussion

Soft tissue sarcomas account for 1% of adult malignancies, while leiomyosarcoma accounts for only 25% of soft tissue sarcomas.^[1,2]^ Primary mediastinal leiomyosarcoma is a rare tumor,^[3]^ usually accounting for <6% of all mediastinal masses, and about 55% of these tumors are malignant, with common types including malignant peripheral nerve tumors, Spindle cell sarcoma, Leiomyosarcoma, and Liposarcoma.^[4]^ Leiomyosarcoma originates from tissues with smooth muscle cells or various mesenchymal cells that have the ability to differentiate into smooth muscle. It is more common in uterus, digestive tract and retroperitoneum, with a few occurring in the vascular wall and rarely in the mediastinum.^[5]^

Leiomyosarcoma of the posterior mediastinum is often difficult to differentiating from neurogenic tumors. Patients may have no symptoms when tumors had small volume. As the tumor grows and progresses, it compresses the esophagus, large blood vessels, or invades the spinal canal, which may lead to swallowing difficulties, thrombosis, breathing difficulties, and a series of compression symptoms. CT and magnetic resonance imaging cannot clearly differentiate leiomyosarcoma and neurogenic tumors, posing a great challenge to clinical physicians in the diagnosis and treatment process. Neurogenic tumors often originate from the sympathetic nervous system and are often located in the posterior mediastinal region adjacent to the spine. They often have a regular shape, uniform density, and clear boundaries. However, leiomyosarcoma generally has a larger volume, irregular shape, and significant mass effect. In addition, the positional relationship between tumors and nerve roots can also help distinguish between these 2 types of diseases. At present, the gold standard for differentiating these 2 types of diseases is still pathological tissue biopsy.^[6]^

Surgical resection is currently the main treatment method for mediastinal leiomyosarcoma. Recurrence is the cause of death for most patients, usually occurring within 2 to 3 years after surgery.^[7]^ Engelhardt et al analyzed 976 patients diagnosed with mediastinal sarcoma in the United States and concluded that patients who underwent complete resection of the lesion showed better 5-year overall survival rates than patients with other treatment (30.1% vs 18.9%, *P* = .002). In addition, compared to vascular sarcoma, patients with leiomyosarcoma have more significant benefits during surgery.^[8]^ Lee DH et al reported a case of posterior mediastinal leiomyosarcoma extending to the spinal canal, in which the diseased tissue was not completely removed, resulting in short-term local recurrence and death.^[9]^ Therefore, comprehensive imaging examinations should be conducted before treatment to clarify the extent of tumor invasion and surgical indications.

Depending on the location, size and aggressiveness of the tumor, thoracotomy may be taken instead of VATS. The lesion is prone to adhesion with the surrounding mediastinal structures, including parietal pleura, pericardium, large blood vessels and part of lung tissue, these structures should also be removed during surgery, which undoubtedly increases the difficulty of the procedure. The prognosis of leiomyosarcoma depends on the size and histological grade of the tumor. Only a few studies had reported this disease, there is still controversy about whether to use adjuvant therapy after surgery. Some studies reported that postoperative treatment may cause adverse reactions.^[10]^

## 4. Conclusion

The posterior mediastinum is a rare site of leiomyosarcoma, and it is difficult to distinguish it from other malignant tumors through imaging and clinical manifestations before surgery. Surgical resection is currently the best method for treating this disease, but there are still doubts about the application of postoperative adjuvant therapy. We hope that our research can help clinical doctors better diagnose and treat such diseases.

## Acknowledgments

This work was supported by Hebei Province Key Research and Development Plan Project (22377790D).

## Author contributions

**Investigation:** Hongzhen Zhao, Lingxin Kong.

**Methodology:** Zhonghui Hu.

**Project administration:** Qingtao Zhao, Guochen Duan.

**Writing – original draft:** Hongzhen Zhao.

**Writing – review & editing:** Wenbo Wu, Guochen Duan.
